# Association between Angiotensin-Converting Enzyme Inhibitors and Lung Cancer—A Nationwide, Population-Based, Propensity Score-Matched Cohort Study

**DOI:** 10.3390/cancers12030747

**Published:** 2020-03-21

**Authors:** Shih-Yi Lin, Cheng-Li Lin, Cheng-Chieh Lin, Wu-Huei Hsu, Chia-Der Lin, I.-Kuan Wang, Chung-Y. Hsu, Chia-Hung Kao

**Affiliations:** 1 Graduate Institute of Biomedical Sciences and School of Medicine, College of Medicine, China Medical University, Taichung 40447, Taiwan; oasisbestonly@yahoo.com.tw (S.-Y.L.); cclin@mail.cmuh.org.tw (C.-C.L.); hsuwh@mail.cmuh.org.tw (W.-H.H.); d6355@mail.cmuh.org.tw (C.-D.L.); ikwang@mail.cmuh.org.tw (I.-K.W.); hsucy63141@gmail.com (C.-Y.H.); 2 Division of Nephrology and Kidney Institute, China Medical University Hospital, Taichung, Taiwan; 3 Management Office for Health Data, China Medical University Hospital, Taichung 40447, Taiwan; orangechengli@gmail.com; 4 College of Medicine, China Medical University, Taichung 40447, Taiwan; 5 Department of Family Medicine, China Medical University Hospital, Taichung 40447, Taiwan; 6 Department of Chest Medicine, China Medical University Hospital, Taichung 40447, Taiwan; 7 Department of Otolaryngology, China Medical University Hospital, Taichung 40447, Taiwan; 8 Department of Nuclear Medicine and PET Center, China Medical University Hospital, Taichung 40447, Taiwan; 9 Department of Bioinformatics and Medical Engineering, Asia University, Taichung 40447, Taiwan; 10 Center of Augmented Intelligence in Healthcare, China Medical University Hospital, Taichung 40447, Taiwan

**Keywords:** angiotensin-converting enzyme inhibitors (ACEIs), lung cancer, air pollutant

## Abstract

Background: Direct evidence of lung cancer risk in Asian users of angiotensin-converting enzyme inhibitors (ACEIs) is lacking. Methods: The ACEI cohort comprised 22,384 patients aged ≥ 18 years with a first prescription of ACEI. The comparison angiotensin receptor blocker (ARB) cohort consisted of age-, sex- and comorbidity-matched patients at a ratio of 1:1. The primary outcome was the incidence of lung cancer, which was evaluated using a proportional hazard model. Results: The overall incidence rates of lung cancer in the ACEI and ARB cohorts were 16.6 and 12.2 per 10,000 person-years, respectively. The ACEI cohort had a significantly higher risk of lung cancer than the ARB cohort (adjusted hazard ratio [aHR]. = 1.36; 95% confidence interval [CI]. = 1.11–1.67). Duration–response and dose–response analyses revealed that compared with patients who did not receive ACEIs, patients who received ACEIs for more than 45 days per year (aHR = 1.87; 95% CI = 1.48–2.36) and patients who received more than 540 defined daily doses of ACEIs per year (aHR =1.80; 95% CI = 1.43–-2.27) had a significantly higher risk of lung cancer. The cumulative incidence of lung cancer was also significantly higher in the ACEI cohort than in the ARB cohort (log-rank test, *p* = 0.002). Conclusions: ACEI use is associated with an increased risk of lung cancer compared with ARB use. Patients using ARBs have a significantly lower risk of lung cancer than non-ARB users.

## 1. Introduction

Angiotensin-converting enzyme inhibitors (ACEIs) cause vasodilation by inhibiting the formation of angiotensin II and ACEIs comprise a critical class of antihypertensive medication indicated for heart failure, asymptomatic left ventricular dysfunction, proteinuria, diabetic nephropathy and postmyocardial infarction [1,2]. Safety concerns regarding the use of ACEIs have been raised, especially their cancer risk [3,4,5]. Hicks et al. reported that the use of ACEIs is associated with a 1.14-fold higher risk of lung cancer compared with the use of angiotensin receptor blockers (ARBs) [6]. However, the latest meta-analysis conducted by Bahaj et al. concluded that no significant association exists between ACEI use and the development of lung cancer [7]. Therefore, the association between ACEIs and lung cancer remains unclear [4,6,7,8,9,10]. Possible reasons for the discrepancies among these studies might be the baseline bias of other comorbidities, insufficient follow-up, confounding effects of other antihypertensive medications and inadequate selection of the control cohort [4,6,9,10,11,12].

A nationwide propensity score-matched cohort study to reduce baseline bias from comorbidities and other antihypertensive medications is required.

In addition, air pollution has gradually become a global concern and is associated with an increased risk of lung cancer [13,14]. When determining the hazardous effects of drugs on lung cancer risk in the real-world setting, exposure to air pollutants should be considered a confounding factor. Furthermore, the variant of insertion or deletion in the ACE gene is significantly different between Asian and Caucasian populations [15]. Hicks et al. reported an association between ACEI use and lung cancer but their study participants were mostly Caucasian. Chiang et al. indicated that ACEI and ARB use is not associated with all-cancer risk in the Taiwanese population [16]. However, the results of Chiang et al. cannot be used to establish whether Asian ACEI users have a higher lung cancer risk because that study had a relatively short follow-up period for cancer latency, did not consider other antihypertensive medications and did not specify lung cancer as a study outcome [16]. Direct evidence of lung cancer risk in Asian ACEI users is lacking. Therefore, we combined data from the National Health Insurance Research Database (NHIRD) and Taiwan Air Quality Monitoring Database (TAQMD) to conduct a propensity score-matched cohort study to determine lung cancer risk in Asian ACEI users.

## 2. Methods

### 2.1. Data Source

The National Health Insurance (NHI) program of Taiwan was established in 1995 and provides universal coverage to over 99% of the residents of Taiwan. The NHIRD is a comprehensive database that includes information on hospitalization, emergency care and medical visits. We conducted this population-based retrospective cohort study using data from the Longitudinal Health Insurance Database (LHID), a subset constructed from original registration files and original claims data in the NHIRD. The LHID contains the data of one million enrollees randomly sampled individuals from the NHIRD. Diagnoses were coded with International Classification of Diseases, Ninth Revision, Clinical Modification (ICD-9-CM) diagnostic codes. The NHIRD ensures the encryption of patient information; therefore, informed consent is not required. This study was approved by the Institutional Review Board of China Medical University (CMUH104-REC2-115[CR-4]). We also used the TAQMD for obtaining information on the air pollutants PM2.5, PM10 and SO_2_. We combined and stratified the LHID and the TAQMD data by linking the residential areas of enrollees with nearby air quality monitoring stations.

### 2.2. Study Population

We enrolled patients aged older than 20 years from 1 January 2000 to 31 December 2012 and divided them into two cohorts: the ACEI cohort and the ARB cohort. The ACEI cohort consisted of patients prescribed ACEIs for at least 28 days, whereas the ARB cohort consisted of patients prescribed ARB for at least 28 days. The date of first ACEI or ARB use during the study period was defined as the index date. Patients who had cancer (ICD-9-CM codes 140–208) during the study period or who had a history of lung cancer before the index date were excluded from this study. To control for confounding effects, we performed 1:1 propensity score matching between the ACEI and ARB cohorts by the following covariates: age, sex, monthly income, urbanization level; diagnosis of hypertension, diabetes, tuberculosis, alcohol-related disease, chronic obstructive pulmonary disease (COPD), chronic liver disease, hyperlipidemia, asthma, stroke, coronary artery disease and rheumatologic disease; use of medication, including α-blockers, β-blockers, potassium-sparing diuretics, thiazides, loop diuretics and calcium channel blockers; and air pollutants. Air pollutant concentrations refer to the daily average concentrations of PM2.5, PM10 and SO_2_.

Lung cancer (ICD-9-CM code 162) was defined as the endpoint of this study. All participants were followed-up from the index date until the date of lung cancer diagnosis, withdrawal from the NHI program or December 31, 2013, whichever occurred first.

### 2.3. Statistical Analysis

To estimate the propensity score, a logistic regression model was used, in which ACEI and ARB use status was regressed on the baseline characteristics listed in Table 1. The distributions of demographic characteristics and clinical comorbidity status were compared between the ACEI and ARB cohorts. Differences were examined using Student’s t-test for continuous variables and the chi-square test for categorical variables. The age, PM2.5 μg/m3 daily average, PM10 μg/m^3^ daily average and SO_2_ ppb daily average distribution is not Gaussian/normal distribution. Therefore, we used nonparametric statistics (Mann-Whitney U test) to test age, PM2.5 μg/m^3^ daily average, PM10 μg/m^3^ daily average and SO_2_ ppb daily average differences between both cohorts. We classified monthly income and urbanization into three and four levels, respectively. Cox proportional hazard regression was used to estimate the adjusted hazard ratio (aHR) and 95% confidence interval (CI) of lung cancer occurrence and the results were further analyzed according to the various dose–response categories. Covariables listed in the Table 1 were included in a multivariable Cox proportional hazards regression model. We further analyzed the dose–response effect among patients using ACEI or ARB. We calculated the average days, average dose and average DDD (defined daily dosages) of ACEI and ARB per year by dividing the total used days or total prescribed dose by the follow-up period. We classified the patients into two subgroups by median. We measured the cumulative incidence of lung cancer in the ACEI and ARB cohorts using the Kaplan–Meier method and we assessed the curve difference using the log-rank test. SAS version 9.4 (SAS Institute Inc., Cary, NC, USA) was used for all data analyses. The two-sided significance level was set at *p* < 0.05.

## 3. Results

We selected 22,384 patients who received ACEI treatment and 22,384 patients who received ARB treatment. Sex and age distributions were similar between the two cohorts. The mean age in both cohorts was approximately 59 years. Significant differences were observed in monthly income and urbanization level between the two cohorts (*p* < 0.05). The ARB cohort was more likely to have coronary artery disease (*p* < 0.05). Regarding the distribution of air pollutants, the daily average concentrations of PM2.5, PM10 and SO_2_ were significantly higher in the ACEI cohort than in the ARB cohort (*p* < 0.05) (Table 1).

The mean follow-up times were 6.33 ± 3.52 years and 6.12 ± 3.47 years in the ARB and ACEI cohorts, respectively. At the end of the study period, the overall incidence rates of lung cancer in the ARB and ACEI cohorts were 12.2 and 16.6 per 10,000 person-years, respectively. After multivariable Cox proportional hazards regression model adjusting for age, sex, comorbidities, medication and air pollutants, a significantly higher risk of lung cancer was observed in the ACEI cohort than in the ARB cohort (aHR = 1.36; 95% CI = 1.11–1.67) (Table 2).

Duration–response and dose–response analyses revealed that compared with patients who did not receive ACEI treatment, patients who received ACEI treatment for more than 45 days per year (aHR = 1.87; 95% CI = 1.48–2.36), patients who received more than 540 mg of ACEIs per year (aHR =1.80; 95% CI = 1.43–2.27) and patients who received more than 50 defined daily doses (DDDs) of ACEIs per year (aHR =1.85; 95% CI = 1.46–2.34) had a significantly higher risk of lung cancer. Compared with patients who did not receive ARB treatment, patients who received ARB treatment for fewer than 200 days per year (aHR = 0.61; 95% CI = 0.47–0.80), patients who received more than 11200 mg of ARB per year (aHR =0.62; 95% CI = 0.50–0.79) and patients who received fewer than 200 DDDs of ARB per year (aHR = 0.63; 95% CI = 0.48–0.81) had a significantly lower risk of lung cancer (Table 3).

**Figure 1:**
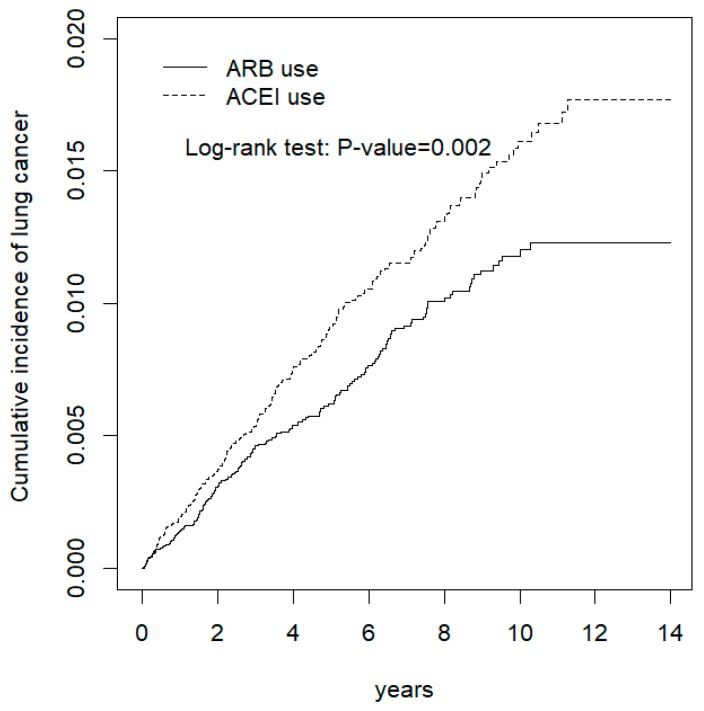
Cumulative incidence of lung cancer between ACEI and ARB users.

In Kaplan–Meier analysis, the cumulative incidence of lung cancer was significantly higher in the ACEI cohort than in the ARB cohort (log-rank test, *p* = 0.002) (Figure 1).

## 4. Discussion

Similar to the findings of Hick et al. [6] our study revealed that ACEI users were at a 1.36-fold higher risk of lung cancer compared with ARB users. Further analysis revealed that ACEI users were at a 1.87-fold and 1.8-fold higher risks of lung cancer when the medication was used for > 45 days or the accumulated dosage of ACEI was > 540 mg, respectively. Patients receiving ARB at an accumulated dosage of > 11,200 mg were at a 0.62-fold lower risk of lung cancer.

In addition to causing vasodilatation in the circulation system, ACEIs are also active in the lungs, where ACEs are abundant [17]. Use of ACEIs could result in increased levels of bradykinin in the lungs, which are normally degraded by ACEIs. This may mediate the sensitization of the airway and enhance the cough reflex [18]. Furthermore, bradykinin is associated with the regulation of neointimal formation and mitogenesis through the mitogen-activated protein kinase pathway [19,20,21]. Chee et al. indicated that bradykinin receptors are highly expressed in the cytoplasm of all types of lung tumors, [22]. which would mediate proangiogenic properties and cancer migration, invasion and metastasis [23]. In addition, ACEI use could cause the accumulation of substance P in the lung. Esteban et al. reported that the activation of neurokinin-1 receptors through substance P is one mechanism linking mitogenesis and cancer promotion and progression [24]. Munoz et al. indicated that substance P may induce the proliferation of both tumor cells and endothelial cells, thus stimulating angiogenesis [25]. Therefore, increased levels of substance P and bradykinin in the lungs may be the mechanism through which ACEI users are at a higher risk of lung cancer. The duration–response and dose–response relationships between ACEI use and lung cancer further strengthen our clinical findings.

In the present study, we observed that ARB users had a significantly lower risk of lung cancer than non-ARB users. The meta-analysis of Zhang et al. also demonstrated that ARBs are associated with significantly lower lung cancer risk [26]. Cohort studies conducted by Chang et al. and Huang et al. have also revealed that ARB use is associated with a decreased risk of lung cancer [11,27]. Bhaskaran et al. observed a 0.84-fold decreased risk of lung cancer in ARB users [28]. Studies have reported that angiotensin II receptors, AT1 receptors and AT2 receptors are involved in enhancing tissue vascular endothelial growth factor protein levels, angiogenesis and tumor growth [29,30]. In addition, AT1 receptors are abundantly expressed in malignant neoplasms, including various types of lung cancer [31,32,33,34]. Fujita et al. revealed that blocking angiotensin II receptors could reduce tumor growth and metastasis [35]. Suganuma et al. and Fujimoto et al. have reported that blockade therapy for angiotensin II receptors could suppress the metastasis of human ovarian cancer and pancreatic cancer in vitro [36,37]. Gong et al. indicated that blocking angiotensin II type 1 receptors could induce apoptotic cell death in human pancreatic cancer cells in vitro [38]. The aforementioned findings indicate the potential biological mechanisms for the finding that ARB users have a lower risk of lung cancer.

Blockade of the renin–angiotensin system (RAS) is well-known to be beneficial in preserving heart and kidney function in those with heart failure, postmyocardial infarction and kidney disease [39]. Therefore, it would be impossible to completely omit all RAS-blocking medications in patients with heart or kidney diseases. Therefore, we employed a study design similar to that of Hicks et al. [6] to compare lung cancer risk in the ACEI and ARB cohorts. We compared lung cancer risk in ACEI and non-ACEI users as well as in ARB users and non-ARB users. Our results directly answer real-world clinical questions and provide sufficient information for decision-making in the selection of RAS blockers.

Several limitations of the current study should be noted. First, information on smoking habits, including intensity and duration; physical exercise; personal exposure to secondhand smoke, asbestos, beryllium, cadmium, silica and other inhaled carcinogens; and body mass index is unavailable in the NHIRD. Since lung cancer was end point of this paper, lack information about smoking habits and exposure to carcinogens of lung cancer should raise concern for baseline bias. However, we have attempted to use COPD as proxy for smoking habitats and we also considered and analyzed asthma, tuberculosis and levels of air pollutants PM2.5, the possible baseline bias might be minimized. Second, we did not adjust for medical visits and chest examination frequency, including chest X-ray, computed tomography, magnetic resonance imaging and ultrasonography; therefore, surveillance bias might be present, because ACEI users have more symptoms of dry cough and undergo more chest examinations. Third, detailed pathological reports such as small cell or non-small cell lung cancer and staging of lung cancer are unavailable in the NHIRD; therefore, further analysis of the association between ACEI use and subgroups of lung cancer could not be performed in this study. Fourth, the mobility of each participant could not be certain. Therefore, select PM2.5 in residential area as a feature would not so representative of real PM2.5 exposure conditions of each participant. Finally, medication adherence could not be fully ascertained in this study.

This study has several strengths. First, we used propensity score matching to mitigate baseline bias between the ACEI and ARB cohorts. Because diverse populations have different reasons for ACEI use, propensity score matching could minimize baseline bias of underlying diseases between the ACEI and ARB cohorts. In addition to matching comorbidities, we also matched each type of antihypertensive medication between the ACEI and ARB cohorts; thereby, baseline bias of antihypertensive medications was minimized. Second, we considered air pollutants (PM2.5, PM10 and SO_2_), which have been recognized as significant risk factors for lung cancer [14,40,41]. According to our literature review, this is the first study considering air pollutants as covariables in the investigation of the association between ACEIs and lung cancer. Third, this study only enrolled those who started using ACEI or ARB from the index date. Those who used ACEIs or ARBs before the index date or those who switched between ACEIs and ARBs were all excluded from our study. Thereby, the bias was considerably minimized. Fourth, NHI covers over 99.7% of the population of Taiwan and contains decades of data. Thus, to the best of our knowledge, the mean follow-up duration of this nationwide population-based study is the longest among similar studies of ACEIs and lung cancer [3,6,42]. This long follow-up enabled a more representative analysis of the relationship between ACEI dosage and duration and lung cancer risk. Furthermore, Hicks et al. speculated that the increased lung cancer risk observed in ACEI users might be due to the protective effect of ARBs against lung cancers [6]. Our study clearly demonstrated the protective effects of ARBs against lung cancer and the potential of ACEIs to cause lung cancer because we separately compared ACEI users and non-ACEI users and ARB users and non-ARB users.

## 5. Conclusions

In conclusion, ACEI users are at a higher risk of lung cancer than ARB users in Taiwan. Dosage–response and duration–response relationships exist between ACEI use and lung cancer risk.

## Figures and Tables

**Table 1:** Demographic characteristics and clinical comorbidity status in study cohorts by propensity score matching.

Covariate	ARB N = 22384		ACEI N = 22384		
	*n*	%	*n*	%	*p*-Value
Age, years					
Mean ± SD ^a^	58.9	13.9	58.8	14.0	0.39
Sex					0.98
Women	10225	45.7	10223	45.7	
Men	12159	54.3	12161	54.3	
Monthly income (NTD) ^†^					0.001
<15,000	6222	27.8	6010	26.9	
15,000−19,999	11791	52.7	12271	54.8	
≥ 20,000	4371	19.5	4103	18.3	
Urbanization level^‡^					0.001
1 (highest)	6666	29.8	6072	27.1	
2	6166	27.6	7005	31.3	
3	3812	17.0	3791	16.9	
4 (lowest)	5740	25.6	5516	24.6	
Comorbidity					
Hypertension	19772	88.3	19776	88.4	0.95
Diabetes	3347	15.0	3320	14.8	0.72
Tuberculosis	434	1.94	450	2.01	0.59
Alcohol-related disease	1199	5.36	1195	5.34	0.93
COPD	3459	15.5	3474	15.5	0.84
Chronic liver disease	5838	26.1	5782	25.8	0.55
Hyperlipidemia	8819	39.4	8631	38.6	0.07
Asthma	3044	13.6	2993	13.4	0.48
Stroke	3148	14.1	3268	14.6	0.11
CAD	8158	36.5	7900	35.3	0.01
Rheumatologic disease	748	3.34	742	3.31	0.87
Medications					
α-Blockers	3359	15.0	3365	15.0	0.94
β-Blockers	13300	59.4	13310	59.5	0.92
Potassium sparing diuretics	3076	13.7	3087	13.8	0.88
Thiazides	10450	46.7	10436	46.6	0.89
Loop diuretics	5783	25.8	5767	25.8	0.86
CCB (non-DHP or DHP)	15484	69.2	15603	69.7	0.22
Others	4173	18.6	4245	19.0	0.38
Air pollutants					
PM2.5 μg/m^3^ daily average (SD)^a^	34.8	8.33	36.0	8.47	< 0.001
PM10 μg/m^3^ daily average (SD)^a^	59.1	13.0	61.1	13.2	< 0.001
SO_2_ ppb daily average (SD)^a^	4.72	1.93	4.82	1.99	< 0.001

Chi-square test, ^a^ Mann-Whitney U test. ^†^ New Taiwan Dollar (NTD), 1 NTD is equal to 0.03 USD. ^‡^ Urbanization level was divided into four levels according to the population density of the residential area, with level 1 being the most urbanized and level 4 being the least. Abbreviations: CAD, coronary artery disease.

**Table 2:** Cox analysis of overall incidence of lung cancer (per 10,000 person-years) and estimated hazard ratios according to medication status.

	ARB	ACEI
Variables	(N = 22384)	(N = 22384)
Person-years	141645	136981
Follow-up time (y), Mean ± SD	6.33 ± 3.52	6.12 ± 3.47
Event, n	173	228
Rate	12.2	16.6
cHR (95% CI)	1(Reference)	1.36(1.11, 1.65) **
aHR (95% CI) ^a^	1(Reference)	1.36(1.11, 1.67) **
		

^a^ Adjusting for sex, monthly income (in NTD), urbanization level and comorbidities including hypertension, diabetes, tuberculosis, alcohol-related disease, COPD, chronic liver disease, hyperlipidemia, asthma, stroke, CAD and rheumatologic disease, medication use including α-Blockers, β-Blockers, potassium-sparing diuretics, thiazides, loop diuretics, CCB (non-DHP or DHP), others and air pollutants including PM2.5, PM10 and SO2. Abbreviations: ACEI, angiotensin II converting enzyme inhibitor; ARB, angiotensin receptor blocker; cHR, crude hazard ratio; aHR, adjusted hazard ratio; ** *p* < 0.01.

**Table 3:** Incidence and adjusted hazard ratios of lung cancer stratified by average days used per year, average dose per year and average DDD (defined daily dosages) per year of angiotensin-converting enzyme inhibitor (ACEI) or angiotensin receptor blocker (ARB) therapy.

Medication Exposed	N	Event	Person-Year	Rate	aHR (95% CI) ^a^
ACEI ^#^					
Non-ACEI	22384	173	141645	12.2	1.00
≤45 days	11159	89	77982	11.4	0.97(0.75, 1.26)
>45 days	11225	139	58998	23.6	1.87(1.48, 2.36) ***
Non-ACEI					1.00
≤540 mg	11183	85	75254	11.3	0.98(0.75, 1.28)
>540 mg	11201	143	61726	23.2	1.80(1.43, 2.27) ***
Non-ACEI					1.00
≤50 DDD	11215	91	77329	11.8	0.99(0.76, 1.28)
>50 DDD	11169	137	59651	23.0	1.85(1.46, 2.34) ***
ARB^#^					
Non-ARB	22384	228	136981	16.6	1.00
≤200 days	11175	78	73104	10.7	0.61(0.47, 0.80) ***
>200 days	11209	95	68541	13.9	0.88(0.69, 1.13)
Non-ARB					1.00
≤11200 mg	5394	56	27325	20.5	1.17(0.86, 1.59)
>11200 mg	16990	117	114320	10.2	0.62(0.50, 0.79) ***
Non-ARB					1.00
≤200 DDD	11363	81	73414	11.0	0.63(0.48, 0.81) ***
>200 DDD	11021	92	68231	13.5	0.87(0.67, 1.11)

^#^ Average days used per year and average DDD dose per year are partitioned into two segments by median. ^a^ Adjusting for sex, monthly income (NTD), urbanization level and comorbidities including hypertension, diabetes, tuberculosis, alcohol-related disease, COPD, chronic liver disease, hyperlipidemia, asthma, stroke, CAD and rheumatologic disease and medication use including α-Blockers, β-Blockers, potassium-sparing diuretics, thiazides, loop diuretics, CCB (non-DHP or DHP), others and air pollutants including PM2.5, PM10 and SO2. Abbreviations: ACEI, angiotensin II converting enzyme inhibitor; ARB, angiotensin receptor blocker; aHR, adjusted hazard ratio; *** *p* < 0.001.
